# A Hybrid Solid‐State Battery with a Panoramic‐Scale Stack of Bulk Electrodes and a Thin‐Film Electrolyte

**DOI:** 10.1002/smll.202600057

**Published:** 2026-04-16

**Authors:** Hyeseong Jeong, Young Jung Kim, Min Kwan Seo, Dongwook Shin, Jae Jin Kim, Jong‐Ho Lee, Sung Soo Shin, Hyoungchul Kim

**Affiliations:** ^1^ Center for Hydrogen Energy Materials Korea Institute of Science and Technology Seoul Republic of Korea; ^2^ Division of Materials Science and Engineering Hanyang University Seoul Republic of Korea; ^3^ Department of Mechanical Engineering Incheon National University Incheon Republic of Korea; ^4^ Department of Mechanical and System Design Engineering Hongik University Seoul Republic of Korea

**Keywords:** all‐solid‐state batteries, co‐sputtering, hybrid batteries, panoramic‐scale layer stacking, thin‐film solid electrolyte

## Abstract

All‐solid‐state batteries (ASSBs) have attracted attention as next‐generation energy storage systems by their thermal stability and higher‐energy‐density potential. However, thick solid electrolytes in conventional ASSBs remain a key bottleneck, simultaneously increasing stack thickness and limiting ion‐transport efficiency. Here, we develop a hybrid ASSB by depositing a thin‐film electrolyte on a bulk anode substrate and stacking a thick cathode sheet. The thin‐film electrolyte was deposited by co‐sputtering with a Li_2_O capping layer. After thermal annealing in Ar, it formed a pure cubic‐phase Li_6.4_La_3_Zr_1.4_Ta_0.6_O_12_ film with a uniform thickness of 2.5 µm and an out‐of‐plane Li‐ion conductivity of 1.91 × 10^−2^ mS cm^−1^ at room temperature. A pore‐gradient, well‐compacted anode substrate (9.01% porosity) was fabricated via high‐speed mixing and cold pressing, followed by thin‐film sputtering deposition and infrared‐based rapid annealing to integrate the bulk substrate and the thin‐film electrolyte. A hybrid ASSB employing a 60 µm‐thick cathode sheet exhibited stable cycles, delivering an initial charge capacity of 102.96 mAh g^−1^ and a discharge capacity of 52.59 mAh g^−1^, while maintaining a robust electrode‐electrolyte interface after cycling. This work demonstrates the successful operation of a hybrid architecture, offering a new design strategy that integrates bulk electrodes with thin‐film electrolytes toward high‐energy‐density ASSBs.

## Graphical abstract

A hybrid all‐solid‐state battery (ASSB) is implemented by integrating a thin‐film electrolyte on a bulk anode, and a thick cathode sheet. A densified anode substrate with suppressed porosity is prepared by cold pressing. Thin‐film electrolyte is fabricated onto the bulk substrate via co‐sputtering and infrared‐based rapid annealing. This panoramic‐scale integrated approach offers practical insights for high‐energy‐density ASSBs.

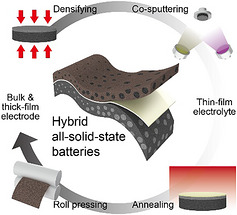

## 1 Introduction

All‐solid‐state batteries (ASSBs) are increasingly recognized as a promising energy storage technology for diverse industrial applications, including electric vehicles (EVs) and energy storage systems (ESSs), owing to their reduced fire‐explosion risk and enhanced thermal stability [1, 2, 3, 4]. Nevertheless, to establish decisive superiority as the next‐generation energy storage solution capable of meeting broad industrial demands, ASSBs must also embody more demanding characteristics such as high energy density, high power output, and large energy capacity [5, 6, 7]. In addition, attaining strong cost competitiveness comparable to that of commercial Li‐ion batteries (LIBs) will be indispensable for broadening the practical applicability of next‐generation ASSBs [8, 9, 10, 11].

In fact, powder‐based bulk processes, which have played a pivotal role in securing the cost competitiveness of commercial LIBs through their convenience and reliability, have been actively employed from the early stages of ASSB development to the present [12, 13, 14]. However, when aiming to achieve ambitious performance targets (i.e., <$80 kWh^−1^, >500 Wh kg^−1^, and >1000 Wh L^−1^) such as those proposed by the U.S. Department of Energy for advanced EV batteries, the inherent limitations of powder processing technologies can impede transformative progress in ASSBs [15, 16, 17]. For example, while the liquid electrolytes account for approximately 24% of the total cell weight in commercial LIBs, solid electrolytes (SEs) constitute nearly 56% in current ASSBs, assuming an SE thickness of 150 µm. This disproportion reduces the fraction of active material available for energy storage, thereby lowering capacity [11, 16]. Furthermore, the presence of a thick SE layer introduces substantial bulk resistance, which restricts high‐power performance [12, 13, 14, 18]. Consequently, to maximize the technological and industrial potential of ASSBs as next‐generation energy storage systems, a paradigm shift beyond conventional materials, architectures, and processing methodologies is urgently required.

Thin‐film battery technology, based on vacuum deposition and microfabrication processes, was first introduced in 1982, marking the inception of this research field [19]. Since the 1990s, advances in thin‐film material deposition—such as the development of LiCoO_2_ electrodes and LiPON (lithium phosphorus oxynitride) SEs—have enabled stable charge–discharge operation in thin‐film batteries [20]. These batteries were subsequently applied to miniaturized power supplies, including portable electronics, implantable medical devices, and microsensor networks [21, 22, 23, 24]. Since 2000, the incorporation of nanostructured materials and flexible substrates has further expanded their potential applications to wearable devices [21, 23]. More recently, research on thin‐film ASSBs employing newly developed SE materials has become increasingly active. Most studies have concentrated on oxide‐based systems, particularly garnet‐type compounds [Li_5_La_3_Nb_2_O_12_, Li_7_La_3_Zr_2_O_12_ (LLZO) and doped LLZO] and perovskite families [Li_0.3_La_0.5_TiO_3_, Li_1.3_Al_0.3_Ti_1.7_(PO_4_)_3_, and Li_1.5_Al_0.5_Ge_1.5_(PO_4_)_3_], which are relatively amenable to vacuum deposition [11, 25, 26]. Their investigations have focused on challenges such as Li volatility, secondary phase formation, crystalline phase transitions, and Li‐ion conductivity (*σ*_Li_) [11, 25, 26, 27, 28, 29]. In contrast, sulfide‐based systems have primarily been explored through thin‐film fabrication using polymer composites or interface engineering, rather than direct thin‐film deposition [30, 31]. Despite decades of progress, thin‐film batteries remain constrained by several critical challenges for next‐generation energy storage devices: (i) low energy capacity due to the limited amount of active material inherent in thin‐film structures, (ii) high manufacturing costs associated with the use of expensive vacuum deposition techniques, and (iii) sluggish ion transport and high interfacial reactivity at the electrode–electrolyte interface [11, 20, 32].

The aforementioned new paradigm of ASSBs centers on integrating the advantages of bulk electrodes, composed of powder‐based active materials, with the electrolyte structures of thin‐film batteries. Although substantial progress has been achieved in individual component technologies, practical demonstrations or evaluations of ASSBs that truly combine thin‐film and bulk approaches remain absent [17, 33, 34]. Realizing this paradigm necessitates the thin‐film fabrication of high*‐σ*_Li_ SE materials, a core technology that effectively reduces bulk resistance and mitigates the specific weight contribution of the SE layer within the cell [11]. Furthermore, in contrast to conventional thin‐film batteries, the direct employment of bulk electrodes is indispensable for attaining practical cell capacities [11, 20, 33, 34]. This highlights the urgent need for next‐generation interfacial technologies capable of mechanically and electrochemically integrating thin‐film electrolytes with bulk electrodes. By enabling the concurrent exploitation of thin‐film and bulk structure advantages, this new paradigm of ASSBs broadens the technology applicability—from compact embedded devices to large‐scale power packs for EVs and ESSs.

In this study, we present a novel hybrid ASSB that integrates a thin‐film LLZO SE layer with bulk electrodes, marking a significant step toward next‐generation energy storage devices. First, the Ta‐doped LLZO (TaLLZO) SE layer was deposited using a vacuum‐based sputtering technique. To satisfy the stringent requirements of the hybrid ASSB—namely high *σ*_Li_, high purity, and scalability—we systematically optimized process parameters, including co‐sputtering, alternating sputtering, and Li_2_O interlayers, and employed a low‐temperature rapid crystallization method. In addition, composite cathode sheets and oxide anode supports composed of powder‐based materials compatible with the thin‐film SE layer were fabricated. In particular, the anode support, serving as the direct substrate for thin‐film SE deposition, was tailored in terms of porosity and microstructure to facilitate uniform thin‐film growth. Finally, by integrating the optimized thin‐film SE layers with bulk electrode structures, both full‐cells and half‐cells were successfully assembled, and their electrochemical performance was comprehensively evaluated at room temperature.

## 2 Results and Discussion

### 2.1 Deposition and Characterization of a TaLLZO Thin Film

**FIGURE 1:**
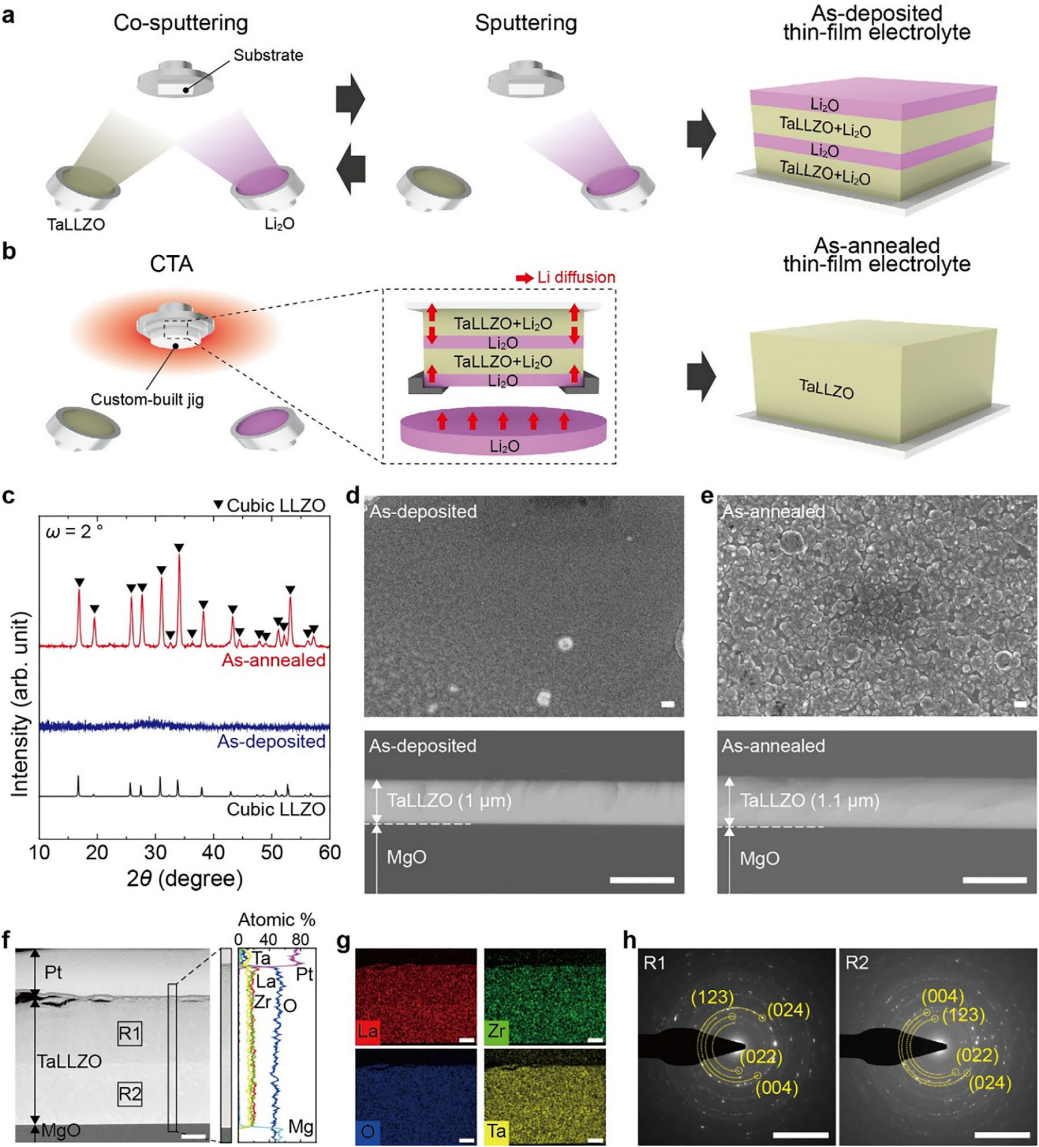
Deposition and characterization of a TaLLZO thin film: (a) deposition of the TaLLZO thin film using co‐ and alternative‐sputtering techniques, (b) CTA in an Ar atomsphere, (c) GIXRD patterns of the TaLLZO thin film in the as‐deposited and as‐annealed states (an offset of 2° was applied), microstructures of the TaLLZO thin film in the (d) as‐deposited and (e) as‐annealed states (all scale bars correspond to 1 µm), (f) TEM cross‐section image with elemental line profiling, (g) elemental TEM‐EDS mapping (all scale bars correspond to 500 nm), and (h) corresponding SAED patterns of individual regions (all scale bars correspond to 5 nm^−1^).

Figure 1 presents the formation process of the TaLLZO thin‐film electrolyte along with the crystallographic and structural characteristics of the resulting film. As schematically illustrated in Figure 1a, the deposition approach employed to fabricate the TaLLZO thin‐film electrolyte is described. Bulk LLZO typically requires high‐temperature annealing above 1100°C to obtain the high*‐σ*_Li_ cubic phase [35, 36]. However, the high volatility of the Li element at elevated temperatures readily induces Li loss, which is particularly pronounced in thin films. This deficiency drives cubic LLZO toward the low*‐σ*_Li_ La_2_Zr_2_O_7_ (LZO) phase [35, 36]. To address this issue, we designed a structural strategy that continuously supplies Li during both deposition and annealing.

Specifically, the proposed design incorporates an internal Li source by co‐sputtering TaLLZO with Li_2_O as the bottom layer, combined with a Li_2_O single‐layer capping on top. This multi‐source/Li_2_O capping configuration provides two major advantages: (i) Li_2_O acts as a diffusible Li reservoir during deposition or subsequent annealing, thereby compensating for Li deficiency in TaLLZO; and (ii) positioning Li sources both within the film and at the top surface mitigates out‐of‐plane Li gradients and compositional inhomogeneity. For the bottom layer, we compared structural differences between two deposition strategies: TaLLZO thin films formed by TaLLZO–Li_2_O co‐sputtering (Figure S1a,b) and those produced by alternating sputtering of TaLLZO and Li_2_O (Figure S1c,d). Scanning electron microscopy (SEM) images reveal that multilayer films exhibit pronounced interlayer gaps in both the as‐deposited and annealed states relative to co‐sputtered films. This phenomenon is attributed to weakened initial interfacial bonding during alternating deposition of heterogeneous thin films, as well as residual stress accumulation arising from mismatched thermal expansion coefficients during annealing, together with non‐uniform densification (Table S1) [37, 38, 39, 40, 41]. Accordingly, we adopted TaLLZO−Li_2_O co‐sputtering for the bottom layer and applied an additional Li_2_O capping layer to ensure sufficient Li compensation.

The phase formation of TaLLZO thin film is highly sensitive to the annealing environment, as exposure to ambient air induces reactions with H_2_O and CO_2_, resulting in the formation of a Li_2_CO_3_ contamination layer on the film surface [42]. Figure 1b illustrates the conventional thermal annealing (CTA) protocol, which integrates an inert atmosphere with an additional Li‐compensation strategy to crystallize the deposited TaLLZO precursor films [42, 43, 44]. CTA was performed in the sputtering chamber under flowing Ar to promote phase formation while minimizing exposure to ambient air. Specifically, a custom‐built jig was employed to provide Li compensation during the annealing step (Figure S2). The jig was designed to position Li_2_O pellets around the thin film, thereby enabling continuous Li diffusion throughout annealing. As a result, this configuration simultaneously suppresses Li volatilization, facilitates compensatory Li diffusion, and prevents air exposure during the annealing step.

Next, the crystallographic structure and phase evolution of the film were examined under varying annealing gas atmosphere (Figure S3). As the O_2_ flow ratio increased relative to Ar, the intensity of LZO peaks progressively strengthened, whereas annealing under pure Ar consistently yielded only the cubic‐LLZO phase. This trend aligns with previous reports indicating that Li volatilization is accelerated in oxidative atmospheres, thereby driving LLZO toward LZO formation [44, 45]. Notably, a highly crystalline cubic‐LLZO phase was successfully obtained even at the relatively low annealing temperature of 650°C (Figure 1c) [44]. Grazing incidence X‐ray diffraction (GIXRD) analysis of the TaLLZO film before and after annealing revealed no distinct peaks in the as‐deposited state, suggesting an amorphous or poorly crystalline film under the present deposition conditions [28]. In contrast, the annealed film exhibited clear diffraction peaks corresponding to the cubic‐LLZO phase (ICSD 422259), confirming effective formation of the cubic structure. These findings indicate that the Li‐compensation strategy outlined in Figure 1a,b extends beyond simple compositional compensation; rather, it stabilizes the thermochemical environment necessary to achieve the targeted cubic phase at reduced temperatures. Furthermore, annealing in an Ar atmosphere minimizes air exposure, thereby supporting a process design that suppresses the nucleation of undesired secondary phases from the earliest stages of structural formation.

Figure 1d,e present the surface and cross‐sectional microstructures of the thin film in both the as‐deposited and as‐annealed states. In the as‐deposited condition, a 1 µm‐thick TaLLZO film was obtained with a flat and uniform morphology. Such thickness uniformity and smooth interfacial characteristics are expected to mitigate localized stress concentrations during annealing and to promote homogeneous Li diffusion and crystallization across the entire film. As shown in Figure S4, 3D surface profile analysis confirmed that the surface roughness of the as‐annealed TaLLZO thin film (*Sa* = 0.093 µm) was slightly higher than that of the as‐deposited film (*Sa* = 0.075 µm). Nevertheless, cross‐sectional SEM revealed a uniform and dense thin film with a thickness of 1.1 µm, indicating that the overall surface structure remains well‐preserved after deposition and annealing. The slight increase in thickness following annealing is attributed to microstructural rearrangement during the transition to a crystalline state, density variations associated with Li diffusion, and redistribution of nanoscale pores [46].

To further verify the compositional integrity of the annealed TaLLZO thin film, Auger electron spectroscopy (AES) analysis was performed (Figure S5 and Table S2). The quantitative elemental ratios obtained from AES were found to be consistent with the target composition of Li_6.4_La_3_Zr_1.4_Ta_0.6_O_12_, indicating that the lithium‐related species introduced during deposition were effectively incorporated into the TaLLZO matrix after annealing. No significant compositional deviation or elemental imbalance was observed within the detection limit of AES, suggesting the absence of residual insulating lithium‐rich phases that could adversely affect ionic transport. This observation is further supported by the transmission electron microscopy (TEM) analysis (Figure 1f–h), which reveals a dense and uniform TaLLZO layer without noticeable secondary phase contrast or interfacial segregation. Cross‐sectional TEM (Figure 1f) confirms a uniform and dense TaLLZO layer without evidence of interfacial delamination or cracking. The corresponding line‐scan profile revealed laterally consistent elemental distributions along the depth direction. Consistently, energy‐dispersive X‐ray spectroscopy (EDS) mapping (Figure 1g) visualized uniform distributions of La, Zr, Ta, and O across the film. Selected area electron diffraction (SAED) patterns acquired from two distinct regions (R1 and R2) are shown in Figure 1h. For cubic LLZO, with its relatively large lattice parameter (approximately 12.983 Å), Bragg's law and Debye–Scherrer diffraction theory predict that numerous (*hkl*) reflections satisfy the diffraction condition within the accessible angular range, resulting in multiple diffraction rings. The assigned rings, including (024), (004), (123), and (022), can be indexed to cubic LLZO [47, 48]. These findings demonstrate that the crystal structure of TaLLZO is faithfully reproduced even at the nanoscale TEM level. Because the process was designed to supply Li via Li_2_O diffusion from both within and near the film, compositional uniformity can be retained during annealing despite Li volatility.

### 2.2 Electrochemical Properties of the TaLLZO Thin‐Film Electrolyte

**FIGURE 2:**
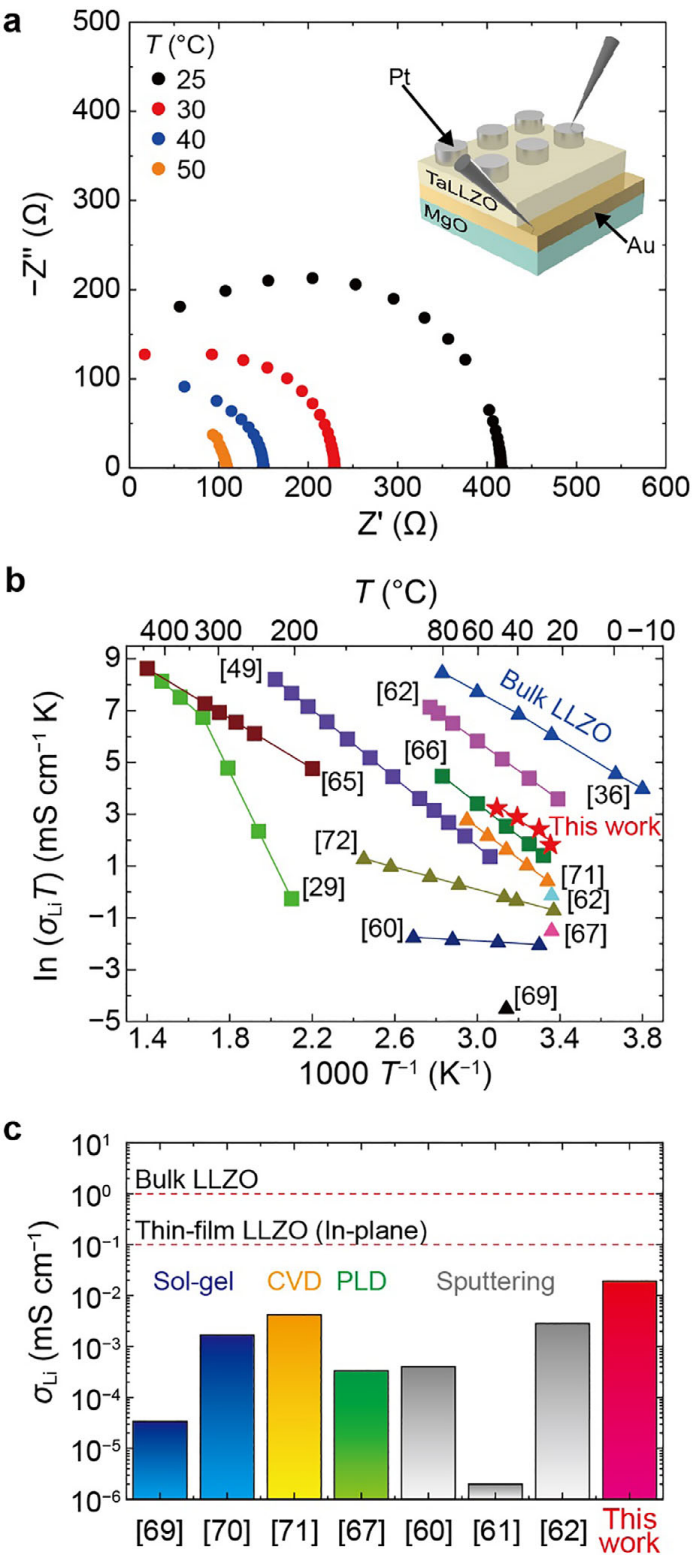
Electrochemical properties of the TaLLZO thin‐film electrolyte: (a) Nyquist plots of the TaLLZO thin film measured at 25°C, 30°C, 40°C, and 50°C (inset: schematic of the Pt/TaLLZO/Au/MgO configuration used for EIS evaluation), (b) comparison of the *σ*_Li_ of the TaLLZO thin‐film electrolyte with values reported in previous studies (detailed information of each result is listed in Table S2), and (c) comparison of the out‐of‐plane *σ*_Li_ of the TaLLZO thin‐film electrolyte as a function of deposition method (CVD and PVD denote chemical vapor deposition and physical vapor deposition, respectively).

Figure 2 presents the electrochemical characteristics of the TaLLZO thin‐film electrolyte fabricated via the optimized process. Because Li‐ion transport in ASSBs predominantly occurs in the out‐of‐plane direction, the out‐of‐plane *σ*_Li_ was evaluated using electrochemical impedance spectroscopy (EIS), and the corresponding activation energy (*E_a_*) was derived. The measurement configuration and equipment used for evaluating the out‐of‐plane ionic conductivity are illustrated in Figure S6. Figure 2a displays Nyquist plots of the TaLLZO thin‐film electrolyte measured at 25°C, 30°C, and 50°C. Thermally stable Au and Pt electrodes were employed as the bottom and top contacts, respectively, on LLZO [49]. As the temperature increased, the semicircle observed in the Nyquist plots progressively decreased in size, indicating reduced overall impedance. To clarify the origin of this change, frequency‐dependent analysis was conducted. The dominant resistance contribution appeared in the high‐frequency region (>10^4^ Hz), which is typically associated with bulk and grain‐boundary transport within the electrolyte layer [50, 51, 52]. With increasing temperature, the peak in this region gradually decreased, indicating enhanced Li‐ion mobility through thermally activated ionic transport. Importantly, no additional resistance feature emerged in the low‐frequency region (<10^2^ Hz), which is generally attributed to electrode‐related processes or interfacial polarization [50, 51, 52]. This suggests that no apparent interfacial degradation occurred within the investigated temperature range. Consistent with this interpretation, the phase‐angle spectra retained similar frequency dependence across the temperature range, while the imaginary impedance (*Z″*) remained near zero in the low‐to‐mid frequency regime and increased only at higher frequencies (Figure S7). The magnitude of *Z″* decreased with increasing temperature, further supporting that the observed impedance reduction primarily originates from improved ionic transport rather than changes in interfacial resistance. Therefore, the contraction of the Nyquist semicircle is mainly attributed to thermally activated Li‐ion conduction within the electrolyte layer, rather than to structural densification or interfacial modification.

Figure S8 presents the Arrhenius plot of the TaLLZO thin‐film electrolyte. The linear dependence of *σ*_Li_ on temperature confirms that Li‐ion transport follows typical Arrhenius behavior over the measured range. The out‐of‐plane *σ*_Li_ reached 1.91 × 10^−2^ mS cm^−1^ at 25°C, with an *E_a_* of 0.45 eV. While bulk LLZO commonly exhibits high *σ*_Li_ on the order of 10^−1^–10^0^ mS cm^−1^, LLZO thin films generally suffer from reduced *σ*_Li_ due to Li volatilization and crystallization constraints [36]. Accordingly, the *σ*_Li_ of the TaLLZO thin film was compared with previously reported bulk and thin‐film LLZO values (Figure 2b; Table S3) [28, 36, 42, 43, 44, 46, 49, 50, 51, 52, 53, 54, 55, 56, 57, 58, 59, 60, 61, 62, 63, 64, 65, 66, 67, 68, 69, 70, 71]. For previously reported thin‐film LLZO electrolytes, *σ*_Li_ values are typically reported in the range of 10^−3^–10^−1^ mS cm^−1^ for in‐plane measurements and 10^−6^–10^−3^ mS cm^−1^ for out‐of‐plane measurements. Therefore, the out‐of‐plane *σ*_Li_ achieved in this study ranks among the higher values reported for LLZO‐based thin films, and the *E_a_* also falls within the commonly observed range for LLZO.

Overall, these results demonstrate that the TaLLZO thin film fabricated in this study achieves comparatively high *σ*_Li_ while maintaining an acceptable Li‐ion migration barrier. Importantly, this *σ*_Li_ value was measured in the out‐of‐plane direction. Because Li‐ion transport in ASSBs is governed by through‐thickness conduction between electrodes, out‐of‐plane performance provides a more direct and practically relevant metric in thin‐film electrolyte studies. Achieving an out‐of‐plane *σ*_Li_ of approximately 10^−2^ mS cm^−1^ therefore, substantially enhances the feasibility of implementing hybrid/thin‐film ASSB architectures employing thin‐film electrolytes. Figure 2c compares the out‐of‐plane *σ*_Li_ as a function of deposition method [60, 61, 62, 67, 69, 70, 71, 72]. Our results reveal enhanced out‐of‐plane *σ*_Li_ compared with LLZO films produced by sputtering and other thin‐film processes, underscoring the effectiveness of the combined Li‐compensation strategy (Li_2_O reservoir and Li_2_O capping) together with the Ar‐atmosphere annealing protocol that suppresses air exposure.

### 2.3 Fabrication of a TaLLZO Thin Film on a Bulk Anode Substrate

**FIGURE 3:**
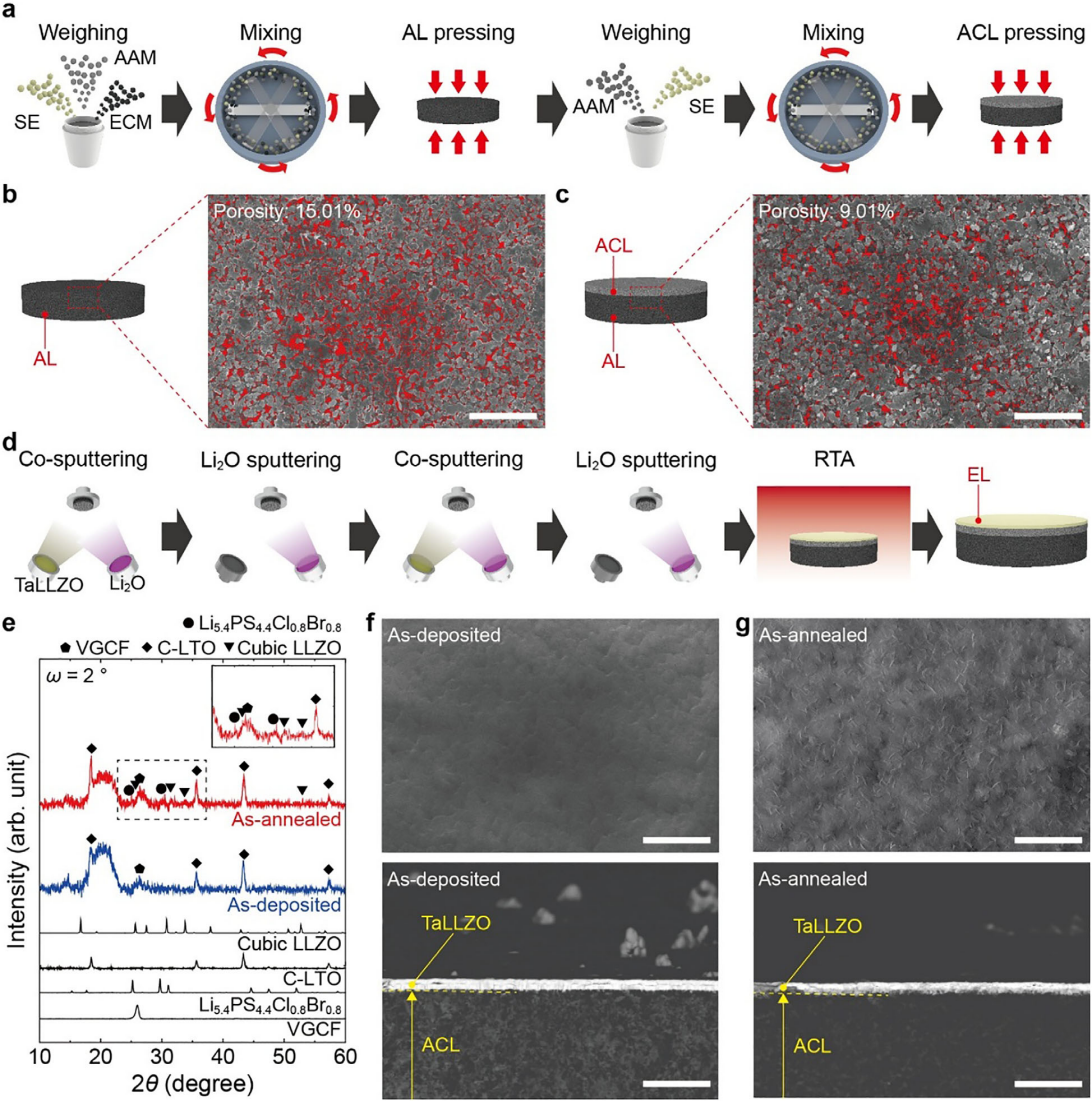
Fabrication and characterization of a TaLLZO thin film on an anode substrate: (a) preparation of a bulk anode substrate with constituent materials, cross‐sectional SEM images of the (b) AL and the (c) ACL surfaces (the red area indicates the pore), (d) schematics on the deposition and RTA processes of the TaLLZO thin film, (e) GIXRD patterns of the bulk anode substrate and TaLLZO thin film in the as‐deposited and as‐annealed states (an offset of 2° was applied), and top‐view and cross‐sectional SEM images of the (f) as‐deposited and (g) as‐annealed TaLLZO thin films. All scale bars correspond to 2 µm.

In conventional ASSBs, bulk‐type and thin‐film‐type architectures each provide distinct advantages: the high capacity of thick electrodes and the low ionic‐transport resistance of thin‐film electrolytes, respectively. To integrate these benefits, we implemented a hybrid device in which a thin‐film electrolyte is inserted between a high‐capacity bulk anode substrate and a thick cathode sheet. By combining the key strengths of both architectures, hybrid ASSBs offer a pathway to simultaneously achieve practicality and high performance. Figure 3 presents a schematic overview of the processes required to realize the hybrid cell, including bulk substrate fabrication, thin‐film electrolyte deposition and annealing, and crystallographic characterization. Direct formation of a thin‐film electrolyte on a bulk substrate in ASSBs requires careful consideration of three critical factors: (i) suppression of thin‐film defects and pinhole formation associated with substrate porosity; (ii) prevention of decomposition reactions or secondary‐phase formation in the substrate (particularly sulfides) during annealing; and (iii) attainment of the target crystalline phase together with a uniform microstructure in the thin‐film electrolyte after annealing.

Figure 3a illustrates the fabrication process of a gradient‐structured bulk anode substrate designed to enable stable thin‐film deposition. First, the anode layer (AL) was prepared by mixing the anode active material [AAM; here, carbon‐coated Li_4_Ti_5_O_12_ (C‐LTO)], SE (Li_5.4_PS_4.4_Cl_0.8_Br_0.8_), and electronic conductive material [ECM; vapor‐grown carbon fiber (VGCF)] via high‐speed mixing, followed by cold pressing at 226 MPa to form a pellet. Subsequently, an anode contact layer (ACL), composed solely of AAM and SE, was strongly compressed at 678 MPa to yield a relatively dense layer. This compact ACL suppresses short‐circuit risks associated with ECM exposure and provides a smoother, denser surface for thin‐film electrolyte deposition. Figure 3b,c compares the surface microstructures and porosities of AL and ACL. The AL exhibits high porosity (15.01%) and protruding carbon‐based ECM features, indicating poor suitability for thin‐film stacking (Figure 3b). In contrast, the ACL shows lower porosity (9.01%) with reduced pore size, thereby providing an effective surface that supports continuous thin‐film growth (Figure 3c).

Figure 3d depicts the deposition of the TaLLZO thin‐film electrolyte layer (EL) directly onto the bulk substrate. In line with the strategy outlined in Figure 1, a TaLLZO−Li_2_O Li‐compensation scheme—combining co‐sputtering with a Li_2_O capping layer—was employed to mitigate Li loss during both deposition and annealing. A key modification, however, was the transition from CTA to rapid thermal annealing (RTA). As demonstrated by the GIXRD analysis of TaLLZO films on the bulk substrate (Figure S9), the original conduction annealing protocol resulted in reduced SE peak intensity within the ACL/AL and induced phase degradation of the thin‐film electrolyte toward LZO [44, 45, 73]. To overcome this limitation, infrared‐based rapid annealing in Ar atmosphere was introduced to shorten high‐temperature exposure, thereby minimizing SE decomposition while promoting thin‐film crystallization. The total annealing duration under rapid annealing was approximately one sixtieth of that in the original process (Figure S10). Consequently, as shown in Figure 3e and Figure S11, the major cubic‐LLZO peaks were clearly observed for films deposited on both ACL and MgO substrates, with no discernible degradation of the ACL/AL constituents. Furthermore, the *σ*_Li_ of the SE exhibited only a slight decrease after rapid annealing (Figure S12; 2.34 mS cm^−1^ as‐prepared vs. 1.78 mS cm^−1^ post‐annealed). Collectively, these results show that rapid annealing successfully reconciles the competing requirements of crystallizing the thin‐film electrolyte while suppressing material degradation. To further examine the possibility of interfacial side reactions during annealing, XPS depth profiling analysis was performed on the TaLLZO films deposited on the ACL substrate. The depth‐dependent spectra revealed that the La_3_ *_d_*, Zr_3_ *_d_*, and O_1_ *_s_* signals remained consistently distributed along the film thickness without noticeable compositional fluctuation or formation of secondary interfacial phases (Figure S13). These observations indicate that no significant interfacial decomposition or diffusion‐induced reaction layer was formed during annealing. Notably, the chemical states of TaLLZO were preserved throughout the film thickness, suggesting that the annealing process did not introduce detectable compositional degradation within the resolution of XPS.

Figure 3f presents the as‐deposited microstructure of the EL on the bulk anode substrate. Cross‐sectional SEM analysis confirms that the EL forms continuously on the ACL, indicating that the relatively lower porosity and more compact surface of the ACL, compared with the AL, effectively facilitates uniform thin‐film growth. Furthermore, as shown in Figure 3g, the EL maintains its continuity even after rapid annealing in an Ar atmosphere, with no evidence of interfacial mechanical defects such as cracking or delamination. Higher‐magnification interfacial images will be provided in conjunction with ASSB operation results. Collectively, these findings establish that a dense, uniform thin‐film electrolyte with the desired crystal structure can be directly fabricated on a bulk anode, thereby providing a practical foundation for ASSB applications.

### 2.4 Fabrication and Characterization of Hybrid ASSB Cells

**FIGURE 4:**
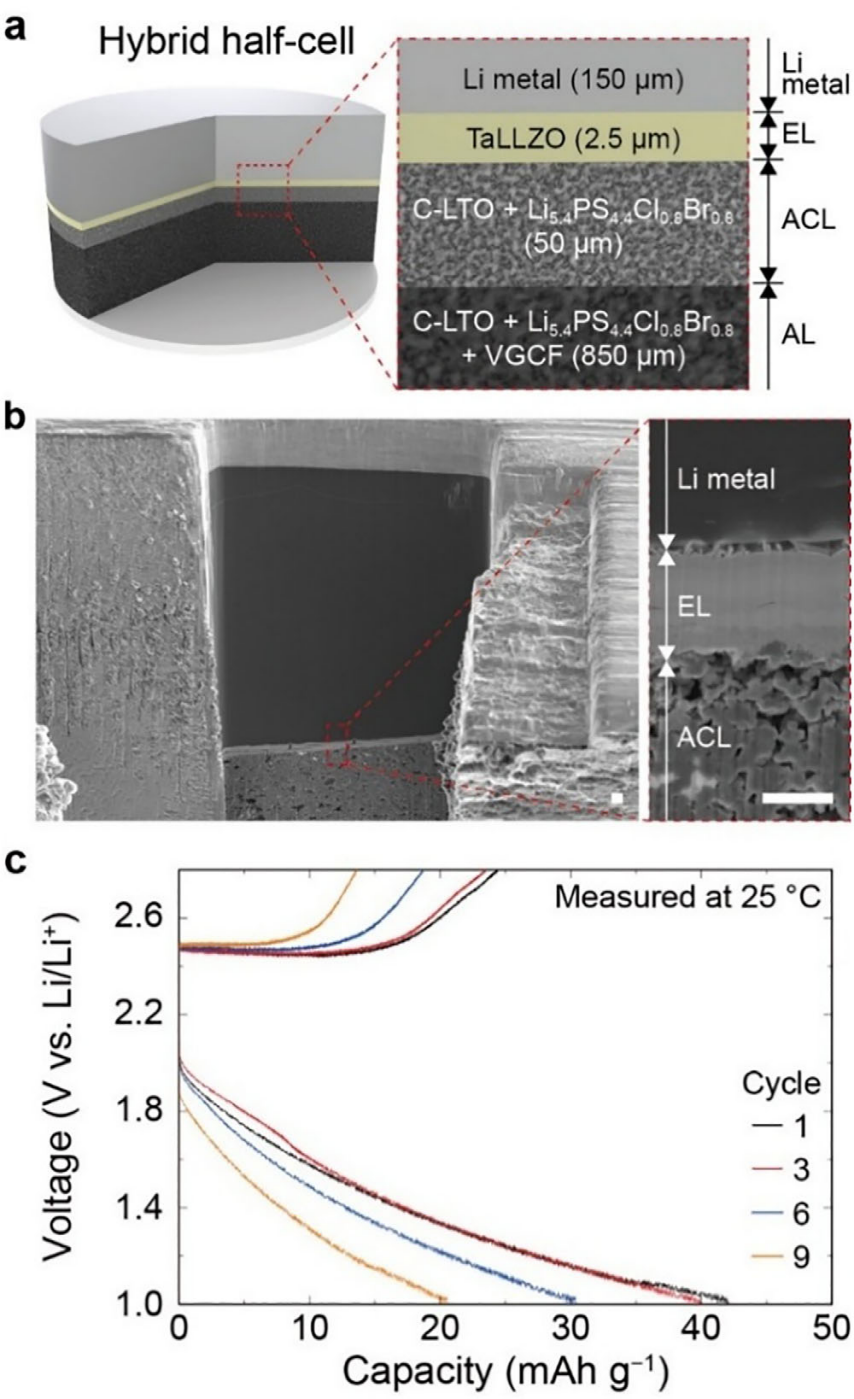
Fabrication and characterization of a hybrid half‐cell: (a) structural schematics illustrating constituent materials and layer thicknesses, (b) sectional SEM images showing the magnified view of the Li/EL/ACL interface (all scale bars correspond to 2 µm), and (c) charge–discharge curves over nine cycles.

To evaluate whether the EL/ACL/AL structure provides (i) the electrochemical characteristics expected of a thin‐film electrolyte and (ii) a suitable interface for charge–discharge operation, a hybrid half‐cell was fabricated by contacting Li metal as the counter electrode under controlled stack pressure. Figure 4a,b illustrate the schematic configuration of the half‐cell and the cross‐sectional focused ion beam (FIB)‐SEM image of the fabricated device, respectively. The TaLLZO thin‐film EL was preserved as a continuous layer between the top and bottom electrodes, with no evidence of short‐circuiting caused by direct electrode contact or penetration. As shown in the charge‐discharge profiles of the half‐cell (Figure 4c), the cell delivered an initial discharge capacity of 42.04 mAh g^−1^ and a charge capacity of 24.39 mAh g^−1^, corresponding to a Coulombic efficiency of 58.02%. Stable cycling performance during 9 cycles was also observed. These results indicate that, despite its micrometer‐scale thickness, the oxide thin‐film electrolyte effectively serves as both a physical and electrical barrier during stacking and operation. Moreover, they confirm that the electrode microstructure and interlayer interfaces were engineered to support reversible electrochemical cycling.

**FIGURE 5:**
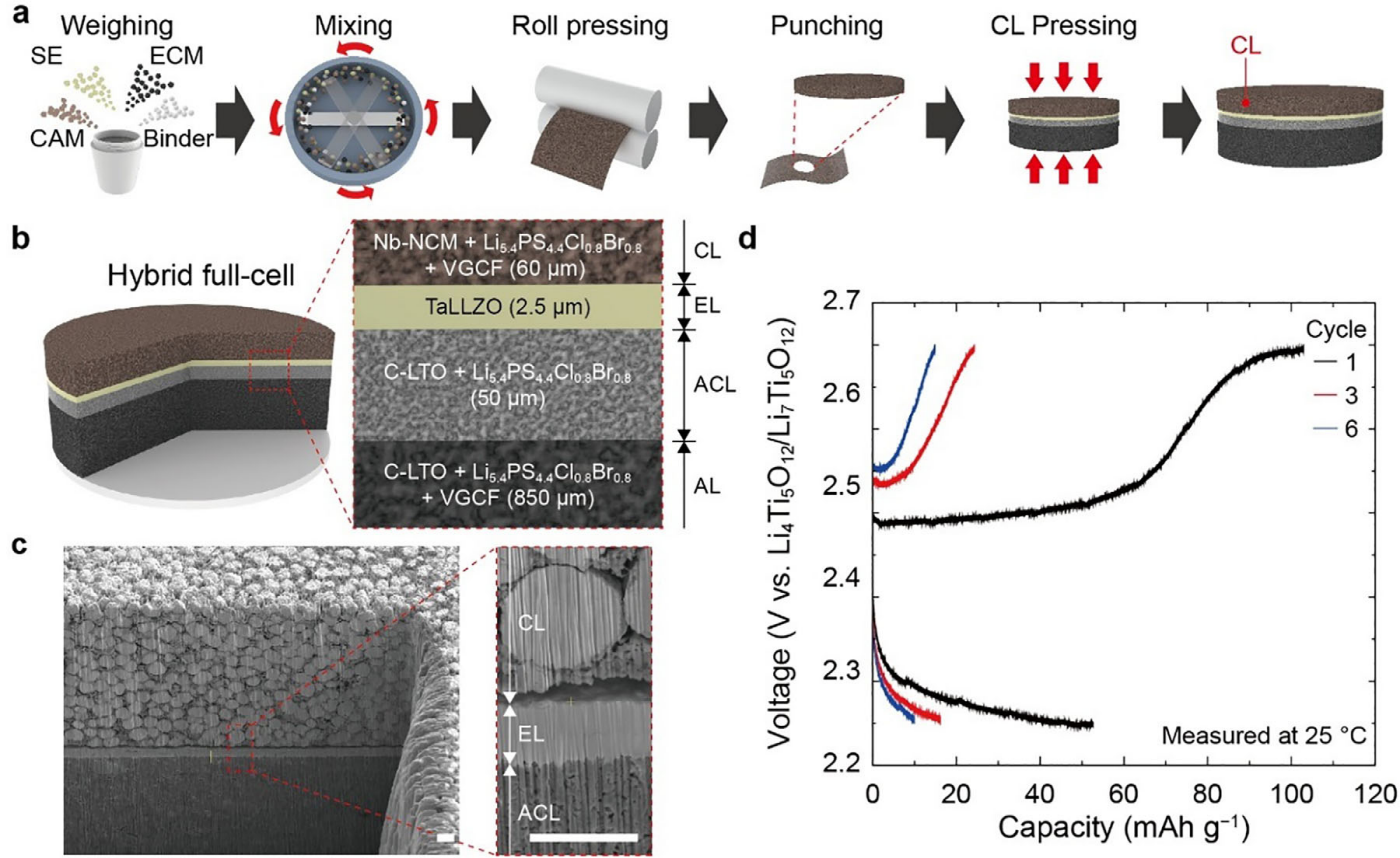
Fabrication and characterization of a hybrid full‐cell: (a) preparation of a thick cathode sheet and stack assembly, (b) structural schematics illustrating constituent materials and layer thicknesses, (c) sectional SEM images showing the magnified view of the CL/EL/ACL interface (all scale bars correspond to 5 µm), (d) charge–discharge curves over six cycles.

To construct a hybrid full‐cell by integrating the thin‐film‐EL/bulk‐AL stack with a high‐capacity cathode layer (CL), a thick cathode sheet architecture was required to simultaneously ensure stable interfacial adhesion and sufficient active‐material loading. Figure 5a shows the fabrication process of the thick cathode sheet. After mixing the cathode active material [CAM; LiNbO_3_‐coated LiNi_0.8_Co_0.1_Mn_0.1_O_2_ (Nb‐NCM)], SE, ECM, and binder [polytetrafluoroethylene (PTFE)], the mixture was roll‐pressed to form a thick sheet. Subsequent uniaxial pressing was applied to enhance CL‐EL adhesion and establish the stacked cell configuration (Figure 5). Figure 5b provides a schematic illustration of the stacking architecture of the hybrid full‐cell. The AL (approximately 850 µm) consists of a C‐LTO‐based composite anode, with a relatively compact ACL (approximately 50 µm) positioned at the TaLLZO‐contacting region. A TaLLZO thin‐film EL (approximately 2.5 µm) was deposited on top, followed by an Nb‐NCM‐based composite CL (approximately 60 µm). The cross‐sectional FIB‐SEM microstructure of the hybrid full‐cell is presented in Figure 5c.

As shown in Figure 5d, the hybrid full‐cell exhibited an initial charge capacity of 102.96 mAh g^−1^ and a discharge capacity of 52.59 mAh g^−1^, corresponding to a Coulombic efficiency of 51.08%. Although these values are lower than the theoretical capacity, they clearly demonstrate that Li insertion and extraction occur within the thick‐CL/thin‐film‐EL/bulk‐AL architecture, thereby enabling electrochemical operation. The key outcome of this work lies in the successful operation of a hybrid cell that integrates bulk electrodes with a thin‐film electrolyte. This demonstration highlights the feasibility of a new architectural paradigm that combines bulk electrode loading, thick composite cathode layers, and a thin‐film electrolyte within a single platform. Such a panoramic‐scale configuration provides a promising pathway to bridge the gap between high‐performance thin‐film electrolytes and practical high‐capacity ASSB designs.

To quantitatively evaluate internal resistances of the hybrid ASSBs, EIS combined with distribution of relaxation times (DRT) analysis was performed for both the hybrid full‐cell and half‐cell (Figure S14 and Table S4). EIS‐DRT enables quantitative extraction of individual resistance components originating from the electrolyte bulk (*R*_bulk_), grain boundaries, and electrode/electrolyte interfaces by deconvoluting overlapping impedance signals as a function of time. The *R*_bulk_ values of the hybrid full‐cell and halfell were comparable, at 177 Ω and 180 Ω, respectively, reflecting the effective reduction of bulk electrolyte resistance achieved by thinning the high‐*σ*_Li_ TaLLZO electrolyte to approximately 2.5 µm. In contrast, the electrode resistance (*R*_electrode_) was determined to be 45 kΩ for the hybrid full‐cell and 38 kΩ for the half‐cell. In both cells, *R*_electrode_ dominated the total resistance, primarily due to interfacial limitations associated with the stack‐pressure assembly designed to minimize mechanical damage to TaLLZO. This finding highlights the need for further interfacial and process optimization. Nevertheless, the successful fabrication and operation of a hybrid ASSB incorporating a uniformly formed micrometer‐scale electrolyte on a bulk anode substrate and a thick cathode sheet underscores the technical significance of this approach. To the best of our knowledge, device‐level demonstrations reporting meaningful performance for such hybrid solid‐state configurations remain scarce. Ultimately, these results establish a new milestone for ASSBs by overcoming the limitations of conventional bulk and thin‐film electrolyte processes and enabling expansion to high‐capacity electrode systems.

## 3 Conclusion

In this study, we demonstrate a hybrid ASSB enabled by a panoramic‐scale film processing strategy, wherein a micrometer‐scale thin‐film electrolyte is directly deposited on a bulk anode substrate and integrated with a thick cathode sheet. To address key challenges in TaLLZO thin‐film processing—namely Li loss and the formation of secondary phases such as LZO or Li_2_CO_3_ upon air exposure—we implemented a Li‐compensation design based on co‐sputtering and insertion of a Li_2_O layer, combined with Ar atmosphere annealing assisted by a Li supply jig. This approach produced a uniform, single‐phase cubic‐LLZO film delivering an out‐of‐plane *σ*_Li_ of 1.91 × 10^−2^ mS cm^−1^ at room temperature with an *E_a_* of 0.45 eV. Furthermore, a compact ACL was introduced, and an infrared‐based rapid annealing was applied to induce electrolyte crystallization while minimizing degradation of the sulfide SE in the anode substrate. A hybrid full‐cell fabricated on these structural and processing foundations operated stably, delivering an initial discharge capacity of 52.59 mAh g^−1^, while maintaining interfacial structure integrity after cycling. EIS‐DRT analysis revealed a relatively low bulk resistance (*R*_bulk_ = 177 Ω), whereas electrode resistance (*R*_electrode_ = 45 kΩ) was dominant, indicating that the overall cell polarization is governed by interfacial charge‐transfer processes rather than ionic transport through the thin‐film electrolyte. Detailed DRT deconvolution further shows that the SE‐CAM interfacial resistance constitutes the largest contribution, leading to constricted Li‐ion transport pathways and a limited effective contact area under moderate stack pressure. In addition, the ion‐transfer barrier can kinetically impede Li‐ion exchange across the interface. These results indicate the importance of optimizing electrode‐electrolyte contact for further performance enhancement. To the best of our knowledge, this work represents the first device‑level demonstration that integrates high‑loading electrodes with a directly deposited thin‑film electrolyte while achieving meaningful ASSB operation. The proposed panoramic‑scale integrated approach provides a pathway to preserve the low‑resistance advantage of thin‑film electrolytes while enabling practical capacity, thereby establishing a foundational technology for scaling next‑generation high‑energy‑density ASSBs. In the present hybrid architecture, the interfacial contact between the cathode layer and the thin‐film electrolyte remains a key factor limiting the overall electrochemical performance. To preserve the mechanical integrity of the layers, the stack was assembled under relatively moderate pressure conditions, which may have prevented the formation of fully intimate interfacial contact. As a result, incomplete physical conformity at the cathode‐electrolyte interface could contribute to the elevated electrode resistance observed in the full‐cell. Future efforts will therefore focus on improving interfacial conformity through process optimization, such as the introduction of compliant interlayers or the use of electrode materials with improved chemical compatibility with LLZO during thermal processing. These approaches are expected to enhance adhesion, reduce interfacial resistance, and ultimately improve the overall cell performance.

## 4 Experimental Section

### 4.1 Thin‐Film Electrolyte Deposition

Thin‐film electrolyte was deposited using a co‐sputtering method with a radio‐frequency (RF) magnetron sputtering system (KVE‐ESC4000, Korea Vacuum Tech). The sputtering targets consist of a main target of Li_6.4_La_3_Zr_1.4_Ta_0.6_O_12_ (Ta‐LLZO; 99.9%, KRTLAB) with a 2‐inch diameter, and a sub‐target of Li_2_O (99.5%, KRTLAB) with a 3‐inch diameter for Li source compensation. Both targets were mounted on a Cu backing plate via In bonding. The target‐substrate (T‐S) distance was fixed at 9 cm for the main target and 11 cm for the sub‐target. Prior to deposition, the chamber interior was evacuated to a low vacuum of 1 × 10^−3^ Torr using a rotary pump, followed by further evacuation to a high vacuum of 2 × 10^−5^ Torr using a turbomolecular pump. Subsequently, the substrate was heated to 300°C at a rate of 10°C min^−1^. During deposition, the substrate was rotated at 5 rpm to ensure uniform film thickness. During the plasma generation step, RF power of 50 and 100 W was applied to the main and sub‐targets, respectively, for 1 min under a high‐purity Ar gas (99.999%) flow of 15 sccm and a working pressure of 20 mTorr. Pressure and gas flow were precisely controlled using a pressure controller (GPC 3000, ATOVAC). The subsequent pre‐sputtering step was conducted for 1 min at the same power under conditions of an Ar gas flow of 20 sccm and a working pressure of 5 mTorr. The main sputtering then proceeded with a four‐layer structure of Li_2_O/TaLLZO/Li_2_O/TaLLZO. The TaLLZO layer was formed by co‐sputtering two targets simultaneously to achieve the desired TaLLZO composition. The Li_2_O layer was deposited using only the Li_2_O target to compensate for the Li source. The deposition time for each layer was fixed at 5 h. The deposition rate was monitored in real‐time using a quartz crystal microbalance sensor.

### 4.2 Thin‐Film Annealing

To form thin‐film TaLLZO with a cubic crystalline phase, the deposited TaLLZO film was annealed using two methods, including Ar atmosphere annealing and Ar atmosphere rapid annealing. The RF magnetron sputtering chamber used for the Ar atmosphere annealing was connected to a glove box system (KK‐021AS, KOREA KIYON), enabling the process without atmospheric exposure. For Li compensation during annealing, 600 mg of Li_2_O powder was compressed at 226 MPa for 1 min to form a pellet with a 13 mm diameter. This Li_2_O pellet and the thin‐film TaLLZO sample were mounted together on a custom‐built jig, which replaced the substrate holder inside the chamber. Subsequently, the chamber was heated to 650°C with an Ar flow of 50 sccm, a working pressure of 20 mTorr, a rotation speed of 5 rpm, and a heating rate of 5°C min^−1^, and then held at this temperature for 2 h. For the Ar atmosphere rapid annealing, the thin film was annealed using a rapid thermal annealing method to form the cubic LLZO phase. Using a rapid thermal annealing processing system (MILA‐5000, ADVANCE RIKO), the samples were annealed at a heating rate of 5°C s^−1^ under an Ar gas atmosphere of 300 sccm, held at 650°C for 2 min.

### 4.3 Characterization of Thin‐Film TaLLZO

To characterize the crystalline phase of the thin‐film TaLLZO deposited on an MgO substrate (MTI Korea) with a size of 10 mm × 10 mm and one side polished in the (100) direction. Grazing incidence X‐ray diffraction (GIXRD) measurements were performed using a thin‐film X‐ray measurement system (DMAX‐2500 PC, Rigaku). The setup included a 0.4 mm divergence slit and a 10 mm divergence horizontal/length slit. The analysis was performed over the 2*θ* range of 10–60° with an increment of 0.04 and a scan speed of 2° min^−1^. An offset angle of 2° was applied to exclude the MgO substrate peak.

To characterize the microstructure, the thin‐film TaLLZO deposited on a non‐conductive MgO substrate was placed in a Pt coater (E‐1045, Hitachi) with the substrate pre‐attached to the holder using carbon tape. Pt was deposited at 15 mA for 10 s to form conductivity between the SEM holder and the samples. The thin‐film TaLLZO sample mounted on the SEM equipment (Regulus 8230, Hitachi) was observed in secondary electrons mode and backscattered electrons mode at an acceleration voltage of 15 kV in top view and cross‐section view.

Cross‐sectional TEM (Talos F200X, Thermo Fisher Scientific) specimens were prepared by FIB milling using a Ga‐ion FIB system (Quanta 3D, Thermo Fisher Scientific). Prior to milling, the sample surface was coated with an Au‐Pd alloy layer. For FIB processing, an electron‐beam‐induced deposition Pt‐C protective layer was first deposited at 5 kV and 0.38 nA, followed by an ion‐beam‐induced deposition Pt‐C protective layer at 30 kV and 0.1 nA. Final thinning was performed at 5 kV and 48 pA. The morphology and microstructure were examined by TEM. The EDS was conducted using the Super‐X EDS system attached to the TEM, operated at an accelerating voltage of 200 kV. Surface roughness was measured by a nanosurface 3D optical profiler (VK‐X3000, KEYENCE).

AES analysis was performed to quantitatively evaluate the elemental composition and stoichiometry of the annealed TaLLZO thin films using a surface analyzer (PHI‐710, ULVAC‐PHI) equipped with a cylindrical mirror analyzer. The primary electron beam energy was set to 10 kV with a target current of 10 nA.

XPS (Nexsa, Thermo Fisher Scientific) analysis was conducted. The base pressure during measurement was maintained at 2.0 × 10^−8^ mbar. A monochromated Al K*α* X‐ray source with a photon energy of 1486.6 eV was used, operating at an accelerating voltage of 12 kV. The beam spot size was set to 400 µm × 400 µm with the sample surface normal to the electron detector. Charge neutralization was achieved using a dual neutralizer consisting of Ar ions and electrons. Wide‐scan spectra were acquired with a pass energy of 200 eV, while high‐resolution spectra were collected using a pass energy of 50 eV. Binding energies were calibrated against the standard C_1_ *_s_* peak at 284.8 eV, and the obtained XPS spectra were subsequently fitted using commercial analysis software (MultiPak, ULVAC‐PHI).

To determine the *σ*_Li_ and *E_a_* of thin‐film TaLLZO, single‐crystal MgO substrates were used. The substrates were cleaned with ethanol prior to use and dried using an air gun. For the formation of the bottom electrode, an Au target (99.99%, DASOM RMS) with a 2‐inch diameter was mounted on a sputtering equipment (KVS‐2002, Korea Vacuum Tech) and deposited for 2 min 30 s under the substrate temperature of 700°C, the Ar gas flow of 5 sccm, the working pressure of 5 mTorr, and the T‐S distance of 50 mm. Then, a TaLLZO thin‐film was deposited onto the Au‐coated MgO substrate and annealed at 650°C for 2 h using an Ar atmosphere annealing. For top electrode formation, a Pt target (99.9%, DASOM RMS) with a 2‐inch diameter was used. Deposition process was performed for 2 min 30 s under the conditions of room temperature, the Ar gas flow of 5 sccm, and the working pressure of 5 mTorr. Pt top electrodes (diameter: 2 mm) were deposited using a shadow mask, defining the effective electrode area used for ionic conductivity measurements. The prepared samples were placed inside a probe station maintained under the Ar gas flow of 200 sccm, and Pt wires were used as current collectors on both electrodes. EIS analysis was performed using electrochemical evaluation equipment (SI‐1287, Solartron Analytical) connected to an impedance/grain‐phase analyzer (SI‐1260, Solartron Analytical), with a 100 mV voltage applied over the frequency range of 5 × 10^6^–1 × 10^−1^ Hz. The Li‐ion conductivity (*σ*_Li_) was calculated using the relation:

$$\sigma_{\mathrm{Li}}=\frac{L}{R\times A}$$

where *L* is the thickness of the TaLLZO thin film, *R* is the resistance obtained from impedance analysis, and *A* is the effective electrode area. The *L* was determined from cross‐sectional SEM analysis, while the *A* was defined by the shadow mask geometry. The *R* was extracted from the high‐frequency intercept of the Nyquist plot. The *E_a_* was calculated based on the Arrhenius equation using the *σ*_Li_ values measured at various temperature.

### 4.4 Fabrication of Composite Anode Substrates

To fabricate the composite anode substrate, C‐LTO as the anode active material, Li_5.4_PS_4.4_Cl_0.8_Br_0.8_ as the SE, and VGCF as the ECM in a 70:25:5 wt.% was mixed using a portable laboratory mill at 20 000 rpm for 150 s. The mixed powder (80 mg) was placed into a 10 mm diameter mold and compressed at 226 MPa for 1 min. Then, an intermediate layer was inserted between the thin‐film TaLLZO layer and the composite anode layer to prevent short circuits caused by VGCF on the pellet surface. To prepare the intermediate layer, C‐LTO and SE were mixed at a 60:40 vol.% ratio using a portable laboratory mill at 20 000 rpm for 150 s. The mixed powder (20 mg) was added to the top side of the composite anode pellet and compressed at 226 MPa for 1 min, then further compressed at 678 MPa for 1 min.

### 4.5 Fabrication of Hybrid All‐Solid‐State Batteries

We deposited the thin‐film TaLLZO onto the composite anode substrate using an RF magnetron sputtering system as Li_2_O/TaLLZO/Li_2_O/TaLLZO structures. The substrate with the deposited thin‐film was annealed by the Ar atmosphere rapid annealing method using rapid thermal annealing equipment to form the cubic LLZO phase while minimizing damage to the sulfide electrolyte. Using an Ar atmosphere rapid annealing, the substrates were annealed at a heating rate of 5°C s^−1^ under an Ar gas atmosphere of 300 sccm, held at 650°C for 2 min.

To fabricate the composite cathode for the hybrid full‐cells, Nb‐NCM was used as CAM. 60 vol.% of CAMs and 40 vol.% of SEs were milled at 20 000 rpm for 160 s using a portable laboratory mill. Following this, 1 wt.% of VGCF was added and milled at 20 000 rpm for 20 s. Finally, 0.9 wt.% of PTFE was added and milled under the same conditions. The mixed powder was kneaded by manual grinding for 10 min. The dough, which was folded to the appropriate size, was shear stressed by roll pressing to form sheets below a thickness of 100 µm. All CAMs and ECMs were dried overnight under vacuum at 120°C to remove moisture. The composite cathode sheet was punched into 6 mm diameter disks and pressed at 470 MPa for 1 min to induce a dense structure. The compressed composite cathode sheet was positioned on top of the TaLLZO, after which the cell was secured using a custom‐built mold apparatus equipped with a pressurized jig to maintain intimate interfacial contact between the electrode and the electrolyte layers. The assembled stack was secured by tightening the nuts on three bolts, and applying a torque force of 2.0 N‐m.

To fabricate hybrid half‐cells, Li metal (thickness: 200 µm) was punched into 6 mm diameter disks, and the surface oxide layer was removed by hand polishing. The polished Li metal was positioned on top of the TaLLZO, and the cell was placed in a custom‐built mold apparatus that is secured using a pressurized jig. The assembled stack was secured by tightening the nuts on three bolts, and applying a torque force of 2.0 N‐m. All cell fabrication procedures were meticulously conducted inside a glove box under an Ar atmosphere, maintaining oxygen levels below 0.5 ppm.

### 4.6 Characterization of Hybrid All‐Solid‐State Batteries

To maintain a constant temperature during electrochemical measurements, all cells were sealed in containers under an Ar atmosphere and placed inside a 25°C incubator (BI‐P‐250, U1Tech). The charge and discharge capacities of the hybrid full‐cell were evaluated at 2.25–2.70 V (vs. Li_4_Ti_5_O_12_/Li_7_Ti_5_O_12_), and those of the hybrid half‐cell at 1.0–2.8 V (vs. Li/Li^+^) using a charge‐discharge system (Model 4300K, Maccor). The current density of 8.8 µA cm^−2^ was applied.

To analyze the crystalline phase changes of the composite anode substrate during the fabrication process, the sample was sealed in Kapton film and mounted on a bulk X‐ray measurement instrument (D8 Advance, Bruker). The measurement was performed in *θ*‐2*θ* mode at a scan speed of 2° min^−1^ over the 2*θ* range of 10–60°. For the phase analysis of the thin‐film TaLLZO deposited on a composite anode substrate, the sample was sealed in Kapton film and measured using GIXRD mode on a thin‐film X‐ray measurement instrument. The settings were a 0.4 mm divergence slit and a 10 mm divergence horizontal/length slit. The analysis was performed over the 2*θ* range of 10–60° with an increment of 0.04 and a scan speed of 2° min^−1^, applying a 2° offsetx angle.

To investigate microstructure, the FIB‐SEM instrument (Helios 5 Hydra UX, Thermo Fisher Scientific) was used. The sample mounted on the FIB‐SEM equipment was milled using an Xe plasma ion source with an accelerating voltage of 30 kV and a current of 2.5 µA under room temperature, and was subsequently observed in cross‐sectional view using the secondary electron mode with an accelerating voltage of 5 kV and a current of 0.4 nA.

For the hybrid full‐cell and hybrid half‐cell, the EIS measurements were performed after charging to 2.5 V and after discharging to 1.3 V, respectively. The obtained EIS spectra were fitted based on an equivalent circuit using ZView software (Scribner Associates). The DRT calculation was based on the Fredholm integral equation. The optimal solution to the equation was derived via the regularization method using time‐frequency domain conversion software (FTIKREG). The quantitative values of the resistance were calculated from the converted DRT spectra based on the derived parameters.

## Funding

This work was supported by 2026 Hongik University Research Fund. This work was also supported by the National Research Foundation of Korea (NRF) grant funded by the Korea government (MSIT) (No. RS‐2024‐00359467).

## Conflicts of Interest

The authors declare no conflicts of interest.

## Supporting information




**Supporting File**: smll73394‐sup‐0001‐SuppMat.docx.

## Data Availability

The data that support the findings of this study are available from the corresponding author upon reasonable request.
