# Supplementary material for: A Hybrid Solid‐State Battery with a Panoramic‐Scale Stack of Bulk Electrodes and a Thin‐Film Electrolyte
Source: Small. 2026 Apr 16;22(44):e00057. doi: 10.1002/smll.202600057 (PMC13450298; doi:10.1002/smll.202600057)
Supplement: Supplementary file 1 [file SMLL-22-e00057-s001.docx]

Supporting Information

A Hybrid Solid-State Battery with a Panoramic-Scale Stack of Bulk Electrodes and a Thin-Film Electrolyte

Hyeseong Jeong, Young Jung Kim, Min Kwan Seo, Dongwook Shin, Jae Jin Kim, Jong-Ho Lee,* Sung Soo Shin,* and Hyoungchul Kim*

**Figure S1.** Microstructural evolution of Ta-doped Li_7_La_3_Zr_2_O_12_ (TaLLZO) thin-films depending on the deposition strategy. (a) Schematic and (b) microstructure of co-sputtering TaLLZO, observed in cross-section view (left) and top view (right). (c) Schematic and (d) microstructure of multilayer deposition TaLLZO, observed in cross-section view (left) and top view (right). All scale bars correspond to 1 μm.

**Figure S2.** Snapshot images of the custom-built jig.

**Figure S3.** Phase evolution of TaLLZO thin-films with varying Ar:O_2_ gas flow-rate ratios. TaLLZO films deposited under (a) Ar:O_2_ = 100:0, (b) 75:25, and (c) 50:50 gas flow conditions. The offset of 2° was applied. LZO denotes La_2_Zr_2_O_7_.

**Figure S4.** Snapshot images and surface profile results of (a) as-deposited and (b) as-annealed TaLLZO films. Surface roughness (*Sa*) was measured by using the embedded software.

**Figure S5.** Auger electron spectroscopy analysis (AES) of as-annealed TaLLZO thin film. (a) Surface morphology of the as-annealed TaLLZO thin film with the regions selected for AES analysis. (b) AES spectra confirming the presence of Li, La, O, Ta, and Zr and indicating a consistent elemental composition across the film surface.

**Figure S6.** Measurement configuration for Li-ion conductivity (*σ*_Li_) of the TaLLZO thin film. (a) A cross-sectional illustration showing the electrode structure for measurement. (b) A photo showing the measuring equipment.

**Figure S7.** Bode plots of the annealed TaLLZO thin film at different temperatures. (a) Phase angle and (b) imaginary impedance as a function of frequency measured at 25, 30, 40, and 50 °C.

**Figure S8.** Arrhenius plot of the thin-film TaLLZO electrolyte. The *σ*_Li_ was measured at 25, 30, 40, and 50 °C. The activation energy (*E_a_*) was calculated as 0.45 eV.

**Figure S9.** Properties of the bulk anode substrate and thin-film TaLLZO after conventional thermal annealing (CTA). (a) Thin-film TaLLZO deposition process. (b) Phase and microstructure in the (c) top view and (d) cross-section view. The offset of 2° was applied. All scale bars correspond to 5 μm. VGCF, EL, C-LTO, and ACL denote vapor-grown carbon fiber, electrolyte layer, carbon-coated Li_4_Ti_5_O_12_, and anode contact layer, respectively.

**Figure S10.** Comparison of thermal annealing processes as a function of time in an Ar atmosphere: (a) rapid thermal annealing (RTA) and (b) CTA.

**Figure S11.** Properties of the thin-film TaLLZO after RTA. (a) Phase and microstructure in the (b) top view and (c) cross-section view. The offset of 2° was applied. All scale bars correspond to 5 μm.

**Figure S12.** Nyquist plots of Li_5.4_PS_4.4_Cl_0.8_Br_0.8_ showing *σ*_Li_ at 25 °C. The *σ*_Li_ of the as-prepared sample is 2.34 mS cm^−1^, while that of the as-annealed sample is 1.78 mS cm^−1^.

**Figure S13.** X-ray photoelectron spectroscopy (XPS) depth profiles of (a) CTA and (b) RTA samples.

**Figure S14.** Electrochemical impedance spectroscopy (EIS) results of hybrid ASSBs using distribution of relaxation times (DRT). (a) Nyquist plot (left) and DRT curve (right) of the full-cell after charge. (b) Nyquist plot (left) and DRT curve (right) of the half cell after discharge.

**Table S1.** Comparison of thermal expansion coefficient and Young’s modulus of TaLLZO and Li_2_O.

| Materials | Thermal expansion coefficient (K^−1^) | Young’s modulus (GPa) | Ref. |
| --- | --- | --- | --- |
| TaLLZO | 1.5×10^−5^ | 154.9 | [1,2] |
| Li_2_O | 2.9×10^−5^ | 108.0 | [3,4] |

**Table S2.** Quantitative atomic composition of annealed TaLLZO thin film obtained by Auger electron spectroscopy analysis. The theoretical atomic percentages are compared with the experimentally measured values.

| Element | Theoretical at.% | Experimental at.% | |
| --- | --- | --- | --- |
|  |  | Measured | Average |
| Li | 27.35 | 32.04 | 30.38 |
| La | 12.82 | 18.62 | 21.74 |
| Zr | 5.98 | 5.03 | 7.93 |
| Ta | 2.56 | 3.37 | 4.02 |
| O | 51.28 | 40.94 | 35.93 |

**Table S3.** Comparison of *σ*_Li_ and *E_a_* of LLZO synthesized by various methods in bulk and thin-film types. PLD, ALD, CVD, AD denote pulsed laser deposition, atomic layer deposition, chemical vapor deposition, and aerosol deposition, respectively.

| Type | Method | Materials | *σ*_Li_ (mS cm^−1^) | | *E_a_* (eV) | Ref. |
| --- | --- | --- | --- | --- | --- | --- |
|  |  |  | Out-of-plane | In-plane |  |  |
| Thin-film | Sputtering | Li_6.4_La_3_Zr_1.4_Ta_0.6_O_12_ | 1.91×10^−2^ | - | 0.45 | This work |
| Bulk | Solid-state-  reaction | Li_6.55_Ga_0.15_La_3_Zr_2_O_12_ | 2.06×10^0^ | - | - | [5] |
|  |  | Li_6.20_Ga_0.30_La_2.95_Rb_0.05_Zr_2_O_12_ | 1.62×10^0^ | - | 0.26 | [6] |
|  | High-temperature sintering | Li_7_Al_0.2−x_Ga_x_La_3_Zr_2_O_12_ | 1.32×10^0^ | - | 0.256 | [7] |
|  |  | Li_6.4_Fe_0.2_La_3_Zr_2_O_12_ | 1.1×10^0^ | - | - | [8] |
|  | Spark plasma  sintering | Li_6.87_La_2.97_Zr_1.6_Ta_0.56_O_12−d_ | 1.35×10^0^ | - | 0.41 | [9] |
|  | Critic acid-  nitrate route | Li_6.55_Ga_0.15_La_3_Zr_2_O_12_ | 1.3×10^0^ | - | 0.3 | [10] |
|  | Chemical  co-precipitation | Li_7_La_3_Zr_2_O_12_ | 2.0×10^−1^ | - | 0.25 | [11] |
| Thin-film | Sputtering | LLZO | 4×10^−4^ | - | 0.70 | [12] |
|  |  | In-doped LLZO | - | 9.6×10^−3^ | - | [13] |
|  |  | Li_7.9_Al_0.2_La_3_Zr_1.6_Ta_0.4_O_12.6_ | 2×10^−6^ | 1.2×10^−1^ | 0.47 | [14] |
|  |  | LLZO | 2.83×10^−3^ | - | - | [15] |
|  |  | Li_6.58_Ga_0.14_La_3_Zr_2_O_12_ | - | 1.6×10^−2^ | 0.35±0.02 | [16] |
|  |  | Al-doped LLZO | - | 1.94×10^−2^ | 0.481 | [17] |
|  |  | Ga-doped LLZO | - | 1.9×10^−1^ | 0.38 | [18] |
|  |  | Li_7_La_3_Zr_2_O_12_  (amorphous) | - | 1.0×10^−4^ | - | [19] |
|  |  | Ga-doped LLZO | - | 6.36×10^−1^ | 0.38±0.02 | [20] |
|  |  | Li_7_La_3_Zr_2_O_12_ | 3.88×10^−4^ | - | 0.51 | [21] |
|  | PLD | Li-La-Zr-O | - | 1.61×10^−3^ | 0.35 | [22] |
|  |  | Al-doped LLZO | - | 1.2×10^0^  (325℃) | 1.39  (below 300℃)  0.60  (above 300℃) | [23] |
|  |  | Ta, Al-doped LLZO  (amorphous) | - | 6.35×10^0^  (400℃) | 0.6 | [24] |
|  |  | Li_7_La_3_Zr_2_O_12_ | - | 1.0×10^−2^ | 0.52 | [25] |
|  |  | Li_7_La_3_Zr_2_O_12_ | 3.35×10^−4^ | - | 0.36 | [26] |
|  |  | Li_7_La_3_Zr_2_O_12_ | - | 2.9×10^−2^ | 0.46±0.03 | [27] |
|  | ALD | Al-doped LLZO | - | 1×10^−5^ | 0.63 | [28] |
|  | Sol-gel | Y-doped LLZO | 3.4×10^−5^ | - | - | [29] |
|  |  | Li_7_La_3_Zr_2_O_12_ | - | 2.4×10^−3^ | 0.52 | [30] |
|  |  | Al-doped LLZO  (amorphous) | 1.67×10^−3^ | - | 0.18 | [31] |
|  | CVD | Li_7_La_3_Zr_2_O_12_ | 4.2×10^−3^ | - | 0.5 | [32] |
|  | AD | Li_6.25_Al_0.25_La_3_Zr_2_O_12_ | - | 1.0×10^−2^ | - | [33] |

**Table S4.** Detailed resistance values obtained from DRT method in hybrid ASSBs. GB, SE, CAM, and SEI refer to grain boundary, solid electrolyte, cathode active material, and solid electrolyte interface, respectively.

|  | Full cell | Half cell |
| --- | --- | --- |
| *R*_bulk_ (Ω) | 177.0 | 180.0 |
| *R*_GB_ (Ω) | 1257.4 | 56.6 |
| *R*_SE-CAM_ (Ω) | 38912.7 | 9806.0 |
| *R*_SEI_ (Ω) | 4463.2 | 27428.6 |
| *R*_SE-Li_ (Ω) | 1058.6 | 1280.8 |

**References**

1.	A.-N. Wang, J.F. Nonemacher, G. Yan, M. Finsterbusch, J. Malzbender, M. Kruger, Mechanical properties of the solid electrolyte Al-substituted Li_7_La_3_Zr_2_O_12_ (LLZO) by utilizing micro-pillar indentation splitting test, *J. Eur. Ceram. Soc.*, 2018, **38**, 3201–3209.

2.	Y. Ren, T. Danner, A. Moy, M. Finsterbusch, T. Hamann, J. Dippell, T. Fuchs, M. Müller, R. Hoft, A. Weber, L.A. Curtiss, P. Zapol, M. Klenk, A.T. Ngo, P. Barai, B.C. Wood, R. Shi, L.F. Wan, T.W. Heo, M. Engels, J. Nanda, F.H. Richter, A. Latz, V. Srinivasan, J. Janek, J. Sakamoto, E.D. Wachsman, D. Fattakhova-Rohlfing, Oxide-Based Solid-State Batteries: A Perspective on Composite Cathode Architecture, *Adv. Energy Mater.*, 2023, **13**, 2201939.

3.	M.C. Billone, Y.Y. Liu, R.B. Poeppel, J.L. Routbort, K.C. Goretta, ELASTIC AND CREEP PROPERTIES OF Li_2_O, *J. Nucl. Mater.*, 1986, **141**, 282–288.

4.	K.P.C. Yao, D.G. Kwabi, R.A. Quinlan, A.N. Mansour, A. Grimaud, Y.-L. Lee, Y.-C. Lu, Y. Shao-Horn, Thermal Stability of Li_2_O_2_ and Li_2_O for Li-Air Batteries: In Situ XRD and XPS Studies, *J. Electrochem. Soc.*, 2013, **160**, A824–A831.

5.	S. Qin, X. Zhu, Y. Jiang, M. Ling, Z. Hu, J. Zhu, Growth of self-textured Ga^3+^-substituted Li_7_La_3_Zr_2_O_12_ ceramics by solid state reaction and their significant enhancement in ionic conductivity, *Appl. Phys. Lett.*, 2018, **112**, 113901.

6.	W. Ku, G. Lee, J.Y. Lee, D.H. Kim, K.H. Park, J. Lim, D. Cho, S.C. Ha, B.G. Jung, H. Hwang, W. Lee, H. Shin, H.S. Jang, J.O. Lee, J.H. Hwang, Rational design of hybrid sensor arrays combined synergistically with machine learning for rapid response to a hazardous gas leak environment in chemical plants, *J. Hazard. Mater.*, 2024, **466**, 133649.

7.	D. Rettenwander, G. Redhammer, F. Preishuber-Pflugi, L. Cheng, L. Miara, R. Wagner, A. Welzl, E. Suard, M.M. Doeff, M. Wilkening, J. Fleng, G. Amthauer, Structural and Electrochemical Consequences of Al and Ga Cosubstitution in Li_7_La_3_Zr_2_O_12_ Solid Electrolytes, *Chem. Mater.*, 2016, **28**, 2384–2392.

8.	D. Rettenwander, R. Wagner, A. Reyer, M. Bonta, L. Cheng, M.M. Doeff, A. Limbeck, M. Wilkening, G. Amthauer, Interface Instability of Fe-Stabilized Li_7_La_3_Zr_2_O_12_ versus Li Metal, *J. Phys. Chem. C*, 2018, **122**, 3780–3785.

9.	S.W. Baek, J.-M. Lee, T.Y. Kim, M.-S. Song, Y. Park, Garnet related lithium ion conductor processed by spark plasma sintering for all solid state batteries, *J. Power Sources*, 2014, **249**, 197–206.

10.	C. Bernuy, W. Manalastas, J.M. Lopez del Amo, A. Aguadero, F. Aguesse, J.A. Kilner, Atmosphere Controlled Processing of Ga-Substituted Garnets for High Li-Ion Conductivity Ceramics, *Chem. Mater.*, 2014, **26**, 3610–3617.

11.	C. Shao, H. Liu, Z. Yu, Z. Zheng, N. Sun, C. Diao, Structure and ionic conductivity of cubic Li_7_La_3_Zr_2_O_12_ solid electrolyte prepared by chemical co-precipitation method, *Solid State Ion.*, 2016, **287**, 13–16.

12.	D.J. Kalita, S.H. Lee, K.S. Lee, D.H. Ko, Y.S. Yoon, Ionic conductivity properties of amorphous Li–La–Zr–O solid electrolyte for thin film batteries, *Solid State Ion.*, 2012, **229**, 14–19.

13.	Z. Yan, Y. Song, S. Wu, Y. Wu, S. Song, X. Wang, Y. Zhu, J. Chen, R. Guo, Y. Xiang, Improving the Ionic Conductivity of the LLZO-LZO Thin Film through Indium Doping, *Crystals*, 2021, **11**, 426.

14.	S. Lobe, C. Dellen, M. Finsterbusch, H.-G. Gehrke, D. Sebold, C.-L. Tsai, S. Uhlenbruck, O. Guillon, Radio frequency magnetron sputtering of Li_7_La_3_Zr_2_O_12_ thin films for solid-state batteries, *J. Power Sources*, 2016, **307**, 684–689.

15.	J. Nong, H. Xu, Z. Yu, G. Zhu, A. Yu, Properties and preparation of Li-La-Ti-Zr-O thin film electrolyte, *Mater. Lett.*, 2015, **154**, 167–169.

16.	M. Rawlence, A.N. Filippin, A. Wackerlin, T.-Y. Lin, E. Cuervo-Reyes, A. Remhof, C. Battaglia, J.L.M. Rupp, S. Buecheler, Effect of Gallium Substitution on Lithium-Ion Conductivity and Phase Evolution in Sputtered Li_7−3x_Ga_x_La_3_Zr_2_O_12_ Thin Films, *ACS Appl. Mater. Interfaces*, 2018, **10**, 13720–13728.

17.	J. Sastre, T.-Y. Lin, A.N. Filippin, A. Priebe, E. Avancini, J. Michler, A.N. Tiwari, Y.E. Romanyuk, S. Buecheler, Aluminum-Assisted Densification of Cosputtered Lithium Garnet Electrolyte Films for Solid-State Batteries, *ACS Appl. Energy Mater.*, 2019, **2**, 8511–8524.

18.	J. Sastre, A. Priebe, M. Dobeli, J. Michler, A.N. Tiwari, Y.E. Romanyuk, Lithium Garnet Li_7_La_3_Zr_2_O_12_ Electrolyte for All-Solid-State Batteries: Closing the Gap between Bulk and Thin Film Li-Ion Conductivities, *Adv. Mater. Interfaces*, 2020, **7**, 2000425.

19.	J. Sastre, M.H. Futscher, L. Pompiz, A. Aribia, A. Priebe, J. Overbeck, M. Stiefel, A.N. Tiwari, Y.E. Romanyuk, Blocking lithium dendrite growth in solid-state batteries with an ultrathin amorphous Li-La-Zr-O solid electrolyte, *Commun. Mater.*, 2021, **2**, 76.

20.	Y. Zhu, S. Wu, Y. Pan, X. Zhang, Z. Yan, Y. Xiang, Reduced Energy Barrier for Li^+^ Transport Across Grain Boundaries with Amorphous Domains in LLZO Thin Films, *Nanoscale Res. Lett.*, 2020, **15**, 153.

21.	S.-Y. Tsai, K.-Z. Fung, Fabrication of garnet solid electrolytes via sputtering for solid-state batteries, *Solid-State Electron.*, 2024, **215**, 108894.

22.	J.S. Park, L. Cheng, V. Zorba, A. Mehta, J. Cabana, G. Chen, M.M. Doeff, T.J. Richardson, J.H. Park, J.-W. Son, W.-S. Hong, Effects of crystallinity and impurities on the electrical conductivity of Li-La-Zr-O thin films, *Thin Solid Films*, 2015, **576**, 55–60.

23.	M. Rawlence, I. Garbayo, S. Buecheler, J.L.M. Rupp, On the chemical stability of post-lithiated garnet Al-stabilized Li_7_La_3_Zr_2_O_12_ solid state electrolyte thin films, *Nanoscale*, 2016, **8**, 14746.

24.	I. Garbayo, M. Struzik, W.J. Bowman, R. Pfenninger, E. Stilp, J.L.M. Rupp, Glass-Type Polyamorphism in Li-Garnet Thin Film Solid State Battery Conductors, *Adv. Energy Mater.*, 2018, **8**, 1702265.

25.	S. Kim, M. Hirayama, S. Taminato, R. Kanno, Epitaxial growth and lithium ion conductivity of lithium-oxide garnet for an all solid-state battery electrolyte, *Dalton Trans.*, 2013, **42**, 13112–13117.

26.	R. Pfenninger, M. Struzik, I. Garbayo, E. Stilp, J.L.M. Rupp, A low ride on processing temperature for fast lithium conduction in garnet solid-state battery films, *Nat. Energy*, 2019, **4**, 475–483.

27.	J. Tan, A. Tiwari, Fabrication and Characterization of Li_7_La_3_Zr_2_O_12_ Thin Films for Lithium Ion Battery, *ECS Solid State Lett.*, 2012, **1**, Q57–Q60.

28.	E. Kazyak, K.-H. Chen, K.N. Wood, A.L. Davis, T. Thompson, A.R. Bielinski, A.J. Sanchez, X. Wang, C. Wang, J. Sakamoto, N.P. Dasgupta, Atomic Layer Deposition of the Solid Electrolyte Garnet Li_7_La_3_Zr_2_O_12_, *Chem. Mater.*, 2017, **29**, 3785–3792.

29.	M. Bitzer, T. Van Gestel, S. Uhlenbruck, B. Hans Peter, Sol-gel synthesis of thin solid Li_7_La_3_Zr_2_O_12_ electrolyte films for Li-ion batteries, Thin Solid Films, 2016, 615, 128–134.

30.	K. Tadanaga, H. Egawa, A. Hayashi, M. Tatsumisago, J. Mosa, M. Aparicio, A. Duran, Preparation of lithium ion conductive Al-doped Li_7_La_3_Zr_2_O_12_ thin films by a sol–gel process, *J. Power Sources*, 2015, **273**, 844–847.

31.	R.J. Chen, M. Huang, W.-Z. Huang, Y. Shen, Y.-H. Lin, C.-W. Nan, Sol-gel derived Li-La-Zr-O thin films as solid electrolytes for lithium-ion batteries, *J. Mater. Chem.*, 2014, **2**, 13277–13282.

32.	C. Loho, R. Djenadic, M. Bruns, O. Clemens, H. Hahn, Garnet-Type Li_7_La_3_Zr_2_O_12_ Solid Electrolyte Thin Films Grown by CO_2_-Laser Assisted CVD for All-Solid-State Batteries, *J. Electrochem. Soc.*, 2017, **164**, A6131–A6139.

33.	J. Han, A. Vu, J.J. Kim, J. Gim, J.R. Croy, T.H. Lee, E. Lee, Room-temperature fabrication of garnet-type solid-electrolyte: Optimizing particle size for high ionic conductivity, *Chem. Eng. J.*, 2024, **481**, 148645.
